# cAMP-Dependent Signaling Pathways as Potential Targets for Inhibition of *Plasmodium falciparum* Blood Stages

**DOI:** 10.3389/fmicb.2021.684005

**Published:** 2021-05-24

**Authors:** Edwin Lasonder, Kunal More, Shailja Singh, Malak Haidar, Daniela Bertinetti, Eileen J. Kennedy, Friedrich W. Herberg, Anthony A. Holder, Gordon Langsley, Chetan E. Chitnis

**Affiliations:** ^1^Department of Applied Sciences, Faculty of Health and Life Sciences, Northumbria University, Newcastle upon Tyne, United Kingdom; ^2^Unité de Biologie de Plasmodium et Vaccins, Département de Parasites et Insectes Vecteurs, Institut Pasteur, Paris, France; ^3^Special Centre for Molecular Medicine, Jawaharlal Nehru University, New Delhi, India; ^4^Laboratoire de Biologie Comparative des Apicomplexes, Faculté de Médecine, Université Paris Descartes – Sorbonne Paris Cité, Paris, France; ^5^INSERM U1016, CNRS UMR 8104, Cochin Institute, Paris, France; ^6^Department of Biochemistry, University of Kassel, Kassel, Germany; ^7^Department of Pharmaceutical and Biomedical Sciences, College of Pharmacy, University of Georgia, Athens, GA, United States; ^8^Malaria Parasitology Laboratory, Francis Crick Institute, London, United Kingdom

**Keywords:** cAMP, PKA, *falciparum*, merozoite, invasion

## Abstract

We review the role of signaling pathways in regulation of the key processes of merozoite egress and red blood cell invasion by *Plasmodium falciparum* and, in particular, the importance of the second messengers, cAMP and Ca^2+^, and cyclic nucleotide dependent kinases. cAMP-dependent protein kinase (PKA) is comprised of cAMP-binding regulatory, and catalytic subunits. The less well conserved cAMP-binding pockets should make cAMP analogs attractive drug leads, but this approach is compromised by the poor membrane permeability of cyclic nucleotides. We discuss how the conserved nature of ATP-binding pockets makes ATP analogs inherently prone to off-target effects and how ATP analogs and genetic manipulation can be useful research tools to examine this. We suggest that targeting PKA interaction partners as well as substrates, or developing inhibitors based on PKA interaction sites or phosphorylation sites in PKA substrates, may provide viable alternative approaches for the development of anti-malarial drugs. Proximity of PKA to a substrate is necessary for substrate phosphorylation, but the *P. falciparum* genome encodes few recognizable A-kinase anchor proteins (AKAPs), suggesting the importance of PKA-regulatory subunit myristylation and membrane association in determining substrate preference. We also discuss how Pf14-3-3 assembles a phosphorylation-dependent signaling complex that includes PKA and calcium dependent protein kinase 1 (CDPK1) and how this complex may be critical for merozoite invasion, and a target to block parasite growth. We compare altered phosphorylation levels in intracellular and egressed merozoites to identify potential PKA substrates. Finally, as host PKA may have a critical role in supporting intracellular parasite development, we discuss its role at other stages of the life cycle, as well as in other apicomplexan infections. Throughout our review we propose possible new directions for the therapeutic exploitation of cAMP-PKA-signaling in malaria and other diseases caused by apicomplexan parasites.

## Introduction

Malaria is caused by apicomplexan parasites of the genus *Plasmodium*. *Plasmodium falciparum*, the most lethal of the species that cause malaria in humans, accounts for almost all the deaths associated with malaria. *P. falciparum* parasites have a complex life cycle during which they infect and multiply within diverse cells and tissues of the human host and mosquito vector. Sporozoites injected into the human host during the bite of an infected female anopheline mosquito travel through the bloodstream to the liver where they invade hepatocytes. The parasites multiply in hepatocytes by schizogony and differentiate into merozoites that are released into the blood stream and go on to invade and multiply within a parasitophorous vacuole inside red blood cells (RBCs) during the asexual blood stage. During this blood stage, some of the parasites transform into gametocytes that are ingested by mosquitoes during a blood meal. The gametocytes undergo gametogenesis in the mosquito midgut in response to environmental signals and form male and female gametes that fertilize to form a zygote. The zygote transforms into a motile ookinete that crosses the midgut wall and forms an oocyst. Each oocyst produces thousands of sporozoites that traverse the hemolymph and invade salivary glands, from where they can be injected by the mosquito to complete the parasite life cycle.

All the clinical symptoms of malaria are associated with the asexual blood stage of the parasite life cycle. Merozoites invade, multiply and egress from host RBCs in repeated cycles, and the rise in parasitemia provokes the clinical symptoms of malaria. The ability of merozoites to invade RBCs is thus key to malaria pathogenesis. RBC invasion is driven by merozoite motility and involves specific molecular interactions between parasite protein ligands and host RBC receptors (Cowman et al., 2017). Many of the parasite proteins that mediate these interactions are located in subcellular apical organelles called micronemes and rhoptries that are the hallmark of apicomplexan parasites (Cowman et al., 2017). The distinctive surface pellicle of these parasites is comprised of the plasma membrane and the inner membrane complex of flattened vesicles lying below the surface membrane, which is the location of the actomyosin motor that drives motility and invasion. Steps such as the timely secretion of microneme and rhoptry proteins in response to external signals as well as activation of the machinery responsible for merozoite motility are key to successful invasion of RBCs by *P. falciparum* merozoites.

Signaling pathways transduce external signals, leading to activation of cellular processes in diverse cells. Transduction of external signals leads to changes in levels of a common set of intracellular second messengers such as calcium (Ca^2+^) and the cyclic nucleotides, cAMP and cGMP (Singh et al., 2010; Dawn et al., 2014; Baker et al., 2017). In *P. falciparum* merozoites, these second messengers activate calcium dependent protein kinases (PfCDPKs), cAMP-dependent protein kinase (PfPKA; catalytic subunit PF3D7_0934800 and regulatory subunit PF3D7_1223100) and cGMP-dependent protein kinase (PfPKG; GeneID: PF3D7_1436600), respectively. The activated kinases phosphorylate specific effector proteins leading to activation of physiological responses. Exposure of *P. falciparum* merozoites to a low K^+^ ionic environment, as found in blood plasma, serves as a signal that raises levels of intracellular cAMP and Ca^2+^ (Singh et al., 2010; Dawn et al., 2014). cAMP is produced in merozoites by soluble adenylyl cyclase β (PfACβ GeneID: PF3D7_0802600) (Dawn et al., 2014; Patel et al., 2019). Mammalian ACβ homologs are usually bicarbonate (HCO_3_^–^) sensitive (Chen et al., 2000) and HCO_3_^–^ levels in cells are generated by carbonic anhydrase (CA), which regulates intracellular pH in cells in response to changes in the external ionic environment (Chen et al., 2000). It has been proposed that changes in intracellular HCO_3_^–^ levels in *P. falciparum* merozoites following exposure to a low K^+^ ionic environment may activate PfACβ and raise cAMP levels (Dawn et al., 2014), resulting in activation of PfPKA (Dawn et al., 2014; Patel et al., 2019). Chemical inhibitors of PfACβ and the catalytic subunit of PfPKA (PfPKAc; GeneID: PF3D7_0934800), as well as inducible knock out of genes encoding PfACβ and PfPKAc, block invasion by *P. falciparum* merozoites (Dawn et al., 2014; Patel et al., 2019), clearly implicating cAMP signaling pathways in RBC invasion.

Transfer of merozoites to a low K^+^ environment also raises the cytosolic level of free Ca^2+^ through a phospholipase C (PLC) dependent pathway (Singh et al., 2010). However, the signal transduction mechanism by which exposure to low K^+^ leads to activation of PLC and raised Ca^2+^ level remains to be conclusively deciphered. The increased Ca^2+^ level activates PfCDPK1 (GeneID: PF3D7_0217500), which has been shown to play a role in invasion (Bansal et al., 2013; Kumar et al., 2017). A similar rise in cGMP levels activates PfPKG, which plays a role in merozoite egress from RBCs containing mature schizonts (Collins et al., 2013b). However, the signal that triggers a rise in cGMP in merozoites remains to be identified.

In this review, we will primarily focus on the role of cAMP and PKA in signaling mechanisms that regulate physiological processes in *P. falciparum* merozoites during RBC invasion. We will also explore how cAMP-dependent signaling pathways could be targeted with small molecule inhibitors to block invasion. Such inhibitors may serve as leads for the development of novel therapeutic strategies to block *P. falciparum* growth.

## cAMP Analogs, Membrane Permeability, and Off-Target Effects

In eukaryotes, the PKA catalytic (C) subunits (Cα, Cβ with several splice variants, Cγ and PrKX) and the functionally non-redundant isoforms of the regulatory subunit (RIα, RIβ, RIIα, and RIIβ) form an inactive PKA holoenzyme complex (R_2_C_2_). Upon consecutive binding of cAMP to the tandem cyclic nucleotide binding (CNB) domains (CNB-A and CNB-B at the C-terminus of the regulatory subunit) the C-subunits are released and can phosphorylate target molecules. In contrast to the mammalian enzyme there is only one R-subunit present in *Plasmodium* PfPKAr or PfPKA-R (GeneID: PF3D7_1223100) (Merckx et al., 2008), a hybrid between RI and RII based on the primary sequence combining features of the human isoforms type I and type II including also the auto-inhibitory sequence of PfPKAr (Haste et al., 2012). Interestingly, the N-terminus of PfPKAr differs significantly from the mammalian counterparts and most likely has specific functions for subcellular localization (Haste et al., 2012). Although detailed biochemical studies are missing, there is evidence that PfPKAr inhibits a PKA activity in parasite extracts in response to cAMP suggesting a similar regulatory mechanism as for mammalian PKA (Merckx et al., 2008).

**FIGURE 1:**
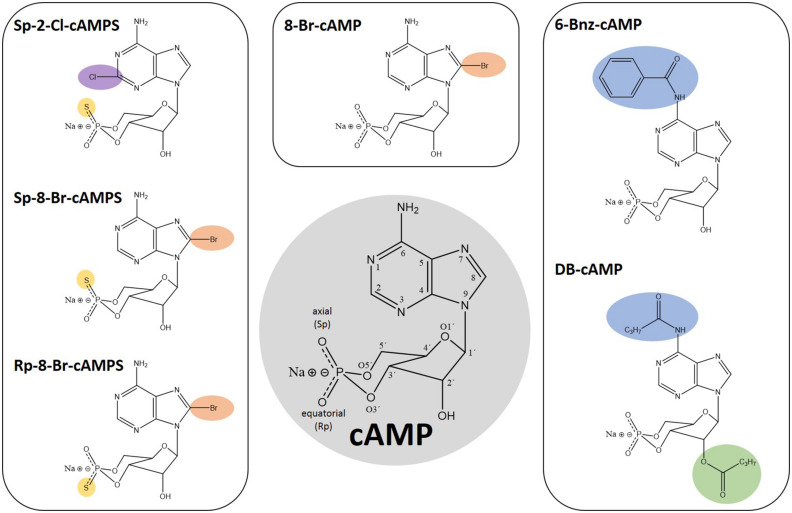
Chemical structures of cAMP and analogs thereof. Numbers in the cAMP structure highlighted in gray indicate the position 1–9 in the adenine ring, 1′–6′ in the sugar moiety as well as the oxygen positions of the cyclic phosphate moiety. The modifications of the depicted analogs are highlighted in color according to their positions: Pos. 2, 6, and 8 in the adenine ring in purple, blue, and orange, respectively. The 2’ position in the sugar moiety is depicted in green and the exchange of exocyclic oxygen to sulfur in the cyclic phosphate moiety is highlighted in yellow.

Given that CNBDs of PfPKAr (CBD) and the human homologs have a sequence identity of about 35% (Littler et al., 2016), PfPKAr specific cAMP analogs (Figure 1) could be a starting point for the development of antimalarial drugs that specifically dysregulate PKA signaling in *P. falciparum*. However, a general limitation of cAMP and its analogs is poor membrane permeability due to their polar ionic structure (Posternak and Weimann, 1974). Furthermore, cAMP is rapidly degraded by phosphodiesterases (Coulson et al., 1983). Dibutyryl-cAMP (DB-cAMP/N6, 2′ O-dibutyryladenosine-3′, 5′-cyclic monophosphate, also known as db-cAMP, Bucladesine and DiB) is a membrane-permeant activator of PKA in mammalian cells (Bartsch et al., 2003; Werner et al., 2011). Already in 1980, DB-cAMP was applied to *Plasmodium* cultures in order to investigate induction of gametocyte formation, and interestingly, DB-cAMP turned out to be only as potent as cAMP (Kaushal et al., 1980), which was surprising, because of the more than 20-fold increased lipophilicity of DB-cAMP compared to cAMP (Braumann and Jastorff, 1985).

Based on the complex membrane structures of a RBC infected with *Plasmodium* (Dearnley et al., 2012), it is conceivable that the permeability of DB-cAMP (Figure 1) differs from that in other cell types, such as platelets, CHO cells, or rat C6 glioma cells (Bartsch et al., 2003; Werner et al., 2011). DB-cAMP is cleaved by intracellular esterases to butyrate and N6-monobutyryl cAMP, activating PKA. However, N6-monobutyryl cAMP may activate PfPKA less efficiently even if present at high concentration in the infected cell. Butyrate has other distinct biological properties and interferes with second messenger signaling pathways. Treatment of *Plasmodium* sporozoites with DB-cAMP initiates motility even more efficiently than the high intracellular cAMP level generated by forskolin (an activator of adenylyl cyclase), or IBMX (an inhibitor of cyclic nucleotide phosphodiesterase) (Kebaier and Vanderberg, 2010). Dawn and co-workers (Dawn et al., 2014) demonstrated that in a parasite line overexpressing PfPKAr (PHL dhfr-PfPKAr), application of DB-cAMP restores both PKA activity and parasite growth. The extracellular addition of DB-cAMP to non-infected RBCs was also used to examine whether the changes in structural and mechanical deformability of non-infected RBCs during *P. falciparum* infection is cAMP-dependent (Paul et al., 2019).

Other membrane permeable cAMP analogs like N6-benzoyl-cAMP (6-Bnz-cAMP/6-Bz-cAMP, Figure 1) and 8-Bromo-cAMP (8-Br-cAMP, Figure 1) have been described as PfPKA activators (Beraldo et al., 2005; Merckx et al., 2008). 6-Bnz-cAMP is known as a selective activator of mammalian PKA with a high lipophilicity, good membrane permeability and increased stability toward phosphodiesterase activity. Interestingly, 6-Bnz-cAMP shows selectivity for the cAMP-binding pocket A of the functionally distinct human PKA regulatory subunit isoforms type I and II (Schwede et al., 2000), which corresponds to PfPKAr CBD1. Treatment of *P. falciparum* cultures with 6-Bnz-cAMP speeds up development to the schizont stage (Beraldo et al., 2005). In the same study, application of 2 mM CaCl_2_ together with 6-Bnz-cAMP increased the cytoplasmic Ca^2+^ concentration in intact parasites, and this increase could be inhibited by PKA specific protein kinase inhibitor (PKI).

Modification of cAMP with bromine (8-Br-cAMP, Figure 1) generates a more lipophilic and thus more membrane-permeant compound. This analog has effects that are comparable with those of DB-cAMP, restoring the growth deficit of transgenic parasites overexpressing PfPKAr (Merckx et al., 2008), even though 8-Br-cAMP is slowly metabolized by phosphodiesterase activity. Phosphorothioate-modified cAMP analogs like Sp-8-Br-cAMPS (8-Bromoadenosine-3′, 5′-cyclic monophosphorothioate, Sp- isomer, Figure 1) have been developed and recently tested on *Plasmodium* by Littler and co-workers (Littler et al., 2016). In contrast to the parent compound 8-Br-cAMP, the axial one of the two exocyclic oxygen atoms in the cyclic phosphate moiety (**“Sp”** stands for the S-isomer corresponding to the R/S nomenclature related to the phosphorus) is modified by **s**ulfur (cAMP**S**). Sp-8-Br-cAMPS is about two-times more lipophilic than 8-Br-cAMP and 4 times more than cAMP, respectively (Schaap et al., 1993; Schwede et al., 2000). Sp-2-Chloro-cAMPS (Sp-2-Cl-cAMPS, Figure 1) is another cAMP-analog with increased lipophilicity and phosphodiesterase stability. Direct comparison between these two analogs and 6-Bnz-cAMP for their capacity to block proliferation of the *P. falciparum* 3D7 asexual blood stage parasite showed a half maximal effect at approximately 4 and 11 μM for Sp-2-Cl-cAMPS and Sp-8-Br-cAMPS, respectively. In contrast, millimolar range 6-Bnz-cAMP had minimal effect. Binding assays with purified PfPKAr revealed affinities of 1.2 nM, 8.9 nM, and 1.3 μM for cAMP, Sp-2-Cl-cAMPS and Sp-8-Br-cAMPS, respectively. Furthermore, Sp-8-Br-cAMPS does not bind CBD2, whereas cAMP and Sp-2-Cl-cAMPS bind with single digit nanomolar affinities to both sites. Crystal structures of PKA-R subunits with the membrane permeable Sp-2-Cl-cAMPS and with cAMP reveal a similar architecture (Littler et al., 2016). However, a unique interaction between PfPKAr Cys-437 in the CBD2 and the C2 position of the adenosine base was discovered, which could be targeted for the development of *Plasmodium* selective cAMP analogs. Thus, novel agonistic (activating) cAMP analogs may be developed to cause uncontrolled premature activation of PfPKAr to impact parasite survival.

Besides the PKA-activating cyclic nucleotide analogs presented here, antagonistic analogs have been developed. In Rp-8-Br-cAMPS (8-BrcAMP-RP isomer, Figure 1) the sulfur modification is at the equatorial position of the cyclic phosphate moiety. In human PKA-R, Rp-8-Br-cAMPS occupies cyclic nucleotide binding sites without causing holoenzyme dissociation. Rp-8-Br-cAMPS is slightly more lipophilic than cAMP and is not metabolized by mammalian phosphodiesterases. In humans, Rp-8-Br-cAMPS discriminates between the functionally distinct PKA regulatory subunit isoform types I and II, preferring type I (Gjertsen et al., 1995) and thus, provides additional selectivity. Rp-8-Br-cAMPS was tested on *Plasmodium* in comparison to PKI and both were able to inhibit the melatonin induced increase of parasitemia by up to 68 and 90%, respectively (Beraldo et al., 2005). Finally, the single parasite EPAC-like gene has been recently genetically demonstrated to be non-essential for parasite growth and merozoite egress by Patel et al. (2019). Therefore, any effect previously observed by Dawn et al. (2014) by Epac agonist (8-pCPT-2’-O-Me-cAMP), and antagonists (ESI-05 ESI-09) may be either due to targeting of an unidentified EPAC-like protein in the parasite that provides redundancy, or due to off-target effects.

More detailed structural studies, for example using nuclear magnetic resonance (NMR) and molecular dynamics simulations, as described recently for PfPKG (Byun et al., 2020), may unravel the distinct conformational changes induced by cyclic nucleotide binding. This may allow the design of high affinity, PfPKAr-selective cyclic nucleotide analogs, which are both membrane permeable and stable against phosphodiesterase degradation.

## PfPKAc as a Target for Development of Anti-Malarial Drugs

PfPKA phosphorylates a range of substrates in merozoites during RBC invasion, and if this activity is essential for asexual blood stage growth, then it is a target for drug development. This conclusion is supported by earlier drug treatment (Syin et al., 2001) and more recent genetic (Patel et al., 2019; Wilde et al., 2019) studies. The identification and use of specific pharmacological inhibitors of PKA activity, together with methods to genetically modify, delete or knockdown the PfPKAc gene, can provide powerful insights into the role of PKA and the signaling pathways in which it is involved. Due to the highly conserved ATP-binding pocket in PfPKAc, commercially available PKA inhibitors have questionable specificity (Murray, 2008) and are unlikely to have greater activity against the parasite than the host enzyme. There is, therefore, a need to develop new inhibitors, for example by biochemical screening of compounds against purified recombinant PfPKAc *in vitro*, as described recently for PfCDPK1 (Ansell et al., 2014). New inhibitors must be able to penetrate into the parasite and must be specific for PfPKA. Accessibility to the enzyme requires that the compound can cross the erythrocyte plasma membrane, the parasitophorous vacuolar membrane and the parasite plasma membrane. The compound must be active at very low concentration as part of the requirement for high specificity. Additional criteria apply to all compounds considered to be lead compounds for further drug development (Burrows et al., 2017). Many kinase inhibitors are ATP-mimetics and the issue of specificity is a major concern, because ATP binding sites may be highly conserved. For example, a series of imidazopyridazine compounds, developed as potent inhibitors of PfCDPK1, were found to also inhibit PfPKG, which has a quite similar ATP binding pocket, and these inhibitors kill parasites through PfPKG inhibition (Green et al., 2015). Other properties of kinases, and regions outside of the ATP binding site may also be targeted for drug development. Post-translational modifications, and the binding of interacting proteins and subunits such as PKAr may be inhibited by small molecules (see previous section). For example, cAMP analogs binding to PKAr perturb the cellular function of PKA (Littler et al., 2016). PKAr is *N*-myristylated and inhibitors of *N*-myristyl transferase (PfNMT; GeneID: PF3D7_1412800) may have a profound effect on PKA function due to loss of membrane binding and subcellular localization signals in their presence (Schlott et al., 2018).

Genetic manipulation of the parasite allows experimental validation of whether the PKAc and PKAr genes are essential, enables the on-target specificity of inhibitors to be substantiated, facilitates a precise dissection of the requirement for particular amino acid residues and permits the use of mutations identified in PKA-specific drug-selected parasites as a key part of structure-function studies (Schlott et al., 2019). In particular, the application of CRISPR-Cas9 methodology enables precise changes to be introduced into the genome. This technology can be coupled with additional tools such as the rapamycin-inducible DiCre system to engineer specific deletions or rearrangements in the PKAc and PKAr genes (Collins et al., 2013a; Jones et al., 2016; Knuepfer et al., 2017), with the glmS ribozyme approach to knockdown expression of the genes (Prommana et al., 2013) and with systems that manipulate protein stability (Armstrong and Goldberg, 2007). Incorporation and expression of a second copy of the PKA genes that can be modified at will, in conjunction with deletion of the endogenous gene enables complementation studies to identify the importance of particular residues in the protein (Koussis et al., 2020). For example, (inducible) replacement of amino acids that are phosphorylated, the introduction of critical changes in the PKAc ATP-binding site, or the replacement of the myristylated N-terminal glycine of PKAr with alanine, would provide key functional insights.

## Targeting Protein–Protein Interactions That Regulate Kinase Activity

Although *P. falciparum* kinases, including PfPKA, have the potential to be targeted using ATP-competitive inhibitors, the kinase domains are conserved and thus share many structural similarities to their human counterparts. Consequently, it remains a major challenge to achieve inhibitor specificity for parasite kinase over host kinase. An alternative strategy is to exploit areas of evolutionary structural divergence to selectively target a *Plasmodium* kinase of interest. Although kinase domains may be conserved across the eukaryotic superfamily, many are flanked by additional domains that serve to regulate the catalytic function of the kinase domain or mediate protein–protein interactions (PPIs) that are critical for kinase-mediated signaling. These regions may be structurally divergent and serve as unique targets for selective inhibition of a kinase of interest.

While PPIs may be attractive for inhibitor development, they are often mediated by interfaces that are shallow and elongated, and therefore cannot be easily disrupted using small molecules. Larger, peptide-based inhibitors have been developed to target these surfaces and interactions, and there are several examples developed to target *Plasmodium* proteins (Helton and Kennedy, 2020). In eukaryotes, a family of A kinase anchoring proteins (AKAPs) interacts with PKA to ensure the correct location for its biological function (Kennedy and Scott, 2015; Bucko and Scott, 2021). A chemically constrained peptide, STAD-2 was designed to mimic a conserved docking helix shared by AKAPs, and which binds directly to the docking/dimerization domain (D/D domain) of human PKA R-subunits (Flaherty et al., 2015). Although no *Plasmodium* AKAP protein has been identified, an AKAP-like protein (GeneID: PF3D7_0512900) was found (Bandje et al., 2016), suggesting that the mechanism of PKA-centered micro-signaling complexes is conserved in parasites. Although the *Plasmodium* AKAP-like protein lacks a homologous D/D domain, it was hypothesized that human AKAPs may play a role in regulating either parasite or human PKA during infection, STAD-2 was added to a culture of *Plasmodium-*infected RBCs (Flaherty et al., 2015) and interestingly, this peptide permeated into infected, but not uninfected, RBCs and could be detected in the parasitophorous vacuole. In addition, the peptide induced lysis of infected RBCs through a PKA-independent mechanism. While this peptide apparently did not disrupt the PPI and AKAP function in *P. falciparum-*infected RBCs, the work demonstrated for the first time that hydrocarbon-stapled peptides can penetrate into intra-erythrocytic *Plasmodium* parasites. Potentially, such peptides may be engineered to target parasite-specific PPIs.

The constrained peptide methodology was then applied to target *Pf*CDPK1, a *Plasmodium* kinase that has been implicated in merozoite invasion (Flaherty et al., 2019). *Pf*CDPK1 contains two additional domains that allosterically regulate its kinase activity, a calmodulin-like domain that acts as a calcium sensor and a Junction-domain (J-domain) that undergoes significant conformational change upon calcium binding. Since the J-domain makes critical contacts with the catalytic domain to allow kinase activation, hydrocarbon-stapled peptides were designed to mimic the J-domain and sequester *Pf*CDPK1 in an allosterically inhibited conformation. The peptide selectively permeated infected RBCs, and was found in late-stage schizonts, inhibiting the targeted kinase and blocking merozoite invasion. This study demonstrates the feasibility of using stapled peptides to target specific parasite proteins and provides a foundation for broadening the repertoire of compound classes to access the parasitophorous vacuole and intraerythrocytic parasite, extending the possible proteins that can be targeted using non-small molecule approaches.

## A Dynamic Signaling Complex Containing PfPKA and PfCDPK1 Is Essential for RBC Invasion and Can Be Targeted to Block Asexual Blood Stage Growth

A rise in cytosolic levels of cAMP and Ca^2+^ in merozoites suspended in a low K^+^ ionic environment that mimics extracellular (EC) ionic conditions, activates PfPKA and PfCDPK1, respectively, which play essential roles in RBC invasion. Interestingly, quantitative phosphoproteomics of merozoites suspended in EC buffer, or in buffer mimicking intracellular (IC) ionic conditions with high K^+^, showed increased phosphorylation of both PfPKAr and PfCDPK1 in EC buffer compared to IC buffer (More et al., 2020). The scaffold protein, 14-3-3, is known to interact with phosphorylated residues of target proteins to assemble protein complexes (Agarwal-Mawal et al., 2003; Kawamoto et al., 2015). Antibodies against Pf14-3-3I (GeneID: PF3D7_0818200) immunoprecipitated Pf14-3-3, PfPKAr and PfCDPK1 from lysates of merozoites suspended in EC buffer suggesting that Pf14-3-3I and phosphorylated PfPKAr and PfCDPK1 form a multi-protein signaling complex (More et al., 2020). In other eukaryotic cells, the subcellular spatio-temporal control of PKAc activity is maintained by interaction with AKAPs. However, AKAP homologs are absent in *P. falciparum* (see above). The function of anchoring PfPKA is mediated by the scaffold protein, Pf14-3-3I (Jain et al., 2020; More et al., 2020). We have not determined the subcellular localization of Pf14-3-3I in merozoites, but previously we localized PfCDPK1 close to the plasma membrane in merozoites (Alam et al., 2015). Since Pf14-3-3I is in a complex with PfCDPK1 and PfPKA it follows that both Pf14-3-3I and PfPKA are also plasma membrane proximal in merozoites.

Importantly, comparing the immunoprecipitates from lysates of merozoites suspended in IC and EC buffers indicated that the protein complex assembles only under EC ionic conditions, therefore assembly of the signaling complex, which is essential for merozoite invasion of RBCs, and is dynamic and only found under conditions that mimic blood plasma. Addition of phosphorylated peptides based on the PfPKAr binding site for Pf14-3-3I to parasite cultures inhibits signaling complex assembly and blocks the microneme secretion that is essential for RBC invasion. Similarly, BV02, a small molecule inhibitor of 14-3-3 interactions in mammalian cells, inhibits Pf14-3-3I interaction with PfPKAr and PfCDPK1 and blocks both microneme secretion and RBC invasion by *P. falciparum* merozoites (More et al., 2020). These studies indicate that targeting the assembly of the Pf14-3-3I-mediated signaling complex may be a viable approach to develop novel therapeutics to block blood stage parasite growth.

## PfPKA Therapeutic Target Proteins Identified From *P. falciparum* Phosphoproteome Datasets

One key question is whether all PKA-mediated phosphorylation has functional significance, or does PKA have promiscuous kinase activity that has no consequence for parasite development? If the phosphorylation of specific substrates, for example in signaling pathways, is essential, those substrates are also potential drug targets. PKA substrates can be identified by comparing the phosphoproteomes of parasites at different stages of development or treated with a PKA-specific inhibitor or with a genetic deletion or knockdown of the PKA gene.

Putative PKA therapeutic targets can be identified by establishing protein kinase – protein substrate relationships from large scale protein phosphorylation data sets. Recent advances in liquid chromatography-tandem mass spectrometry have enabled large scale phosphoproteome screening of various *P. falciparum* life cycle stages, which has resulted in the detection of around 27,200 unique phosphorylation sites in proteins of intracellular asexual blood stage parasites (Solyakov et al., 2011; Treeck et al., 2011; Lasonder et al., 2012; Pease et al., 2013, 2018; Collins et al., 2014; Alam et al., 2015; Kumar et al., 2017; Flueck et al., 2019; Patel et al., 2019) and 2,250 unique phosphorylation sites in proteins of extracellular merozoites (Lasonder et al., 2015; More et al., 2020).

Patel et al. (2019) studied directly the PKA-substrate relationship during the asexual erythrocytic cycle by a quantitative phosphoproteome analysis, comparing wild type parasites with transgenic parasites in which genes encoding PfPKAc and PfACβ (that synthesizes cAMP) were deleted using an inducible recombinase system. The study reported a list of 61 high confidence PfPKA substrate phosphorylation sites (Supplementary Table 1) from the intersection of three quantitative phosphoproteomes (Flueck et al., 2019; Patel et al., 2019). The PfPKA substrate sites have a common feature: the levels of phosphorylation of the site in parasites deficient in PfPKAc and PfACβ were reduced compared to wild type phosphorylation levels as a result of lost kinase activity, and the phosphorylation sites in parasites deficient in phosphodiesterase β (PfPDEβ, GeneID: PF3D7_1321500; a master down-regulator of PfPKA) were upregulated compared to wild type phosphorylation levels by the hyperactivation of PfPKA activity. The set of phosphorylation sites upregulated in PfPDEβ conditional knock down parasites was obtained from previous work (Flueck et al., 2019). The majority (46/61) of the phosphorylation sites contained the broad *P. falciparum* PKA consensus phosphorylation motif (Flueck et al., 2019), which included one or more basic residues at positions −2, −3, and −4 relative to the phosphorylated amino acid (Supplementary Table 1).

More et al. (2020) studied differences in *P. falciparum* protein phosphorylation levels in intra-erythrocytic merozoites prior to egress from the infected cell versus extracellular merozoites competent for red blood cell invasion. Switching merozoites from IC to EC conditions leads to a surge in cAMP concentration and activation of PfPKA, promoting merozoite invasion of RBCs (Dawn et al., 2014). EC conditions are characterized by a low K^+^ concentration that also triggers an increase in Ca^2+^ levels in merozoites, and thereby activation of Ca^2+^-dependent protein kinases such as, but not only, PfCDPK1 (Fang et al., 2018). More et al. (2020) reported that 196 phosphorylation sites (Supplementary Table 2) are upregulated in EC conditions, and that this increase was not blocked by the Ca^2+^ chelator BAPTA-AM. Thus, increased phosphorylation at these sites likely arose directly or indirectly from increased PfPKA activity and not activity of Ca^2+^-dependent kinases. That said, it’s also likely that protein kinases other than PfPKA are also activated upon switching merozoites from IC to EC buffer containing BAPTA-AM. Therefore, an additional stringency filter was added by requiring that the phosphorylation sites are located within the broad PKA consensus sequence motif, and this restriction reduced the number of phosphorylation sites to 71, identifying 35 proteins as potential PfPKA therapeutic targets (Supplementary Table 2).

We also exploited the ensemble of phosphoproteome data (Treeck et al., 2011; Lasonder et al., 2012, 2015; Pease et al., 2013, 2018; Collins et al., 2014; Alam et al., 2015; Kumar et al., 2017; Flueck et al., 2019; Patel et al., 2019; More et al., 2020) to identify novel potential PKA therapeutic targets by (1) establishing putative substrate-PKA relationships from phosphorylation site sequence motifs and by (2) requiring a putative protein - protein interaction between substrate and PKA to be present, as defined in the STRING database. We had previously established the sequence: Arg or Lys followed by any two residues immediately upstream of the phosphorylated Ser or Thr [(R/K)xx(pS/pT)], as the core consensus motif that was most abundant in PKA substrates found in a *P. falciparum* merozoite phosphoproteome (Lasonder et al., 2015). A list of potential PKA substrates was compiled by extracting 5,735 phosphorylated peptide sequences encompassing this PKA consensus motif from the data reported in 12 *P. falciparum* phosphoproteome studies (Supplementary Table 3). The PfPKA consensus motif sequence (R/K)xx(pS/pT) is 30-times more abundant in the two merozoite phosphoproteomes (Lasonder et al., 2015; More et al., 2020) compared to the phosphoproteomes of intracellular asexual blood stages (Solyakov et al., 2011; Treeck et al., 2011; Lasonder et al., 2012; Pease et al., 2013, 2018; Collins et al., 2014; Alam et al., 2015; Kumar et al., 2017; Flueck et al., 2019; Patel et al., 2019) suggesting an important role for PfPKA phosphorylation of these sites in proteins during merozoite invasion of erythrocytes.

**FIGURE 2:**
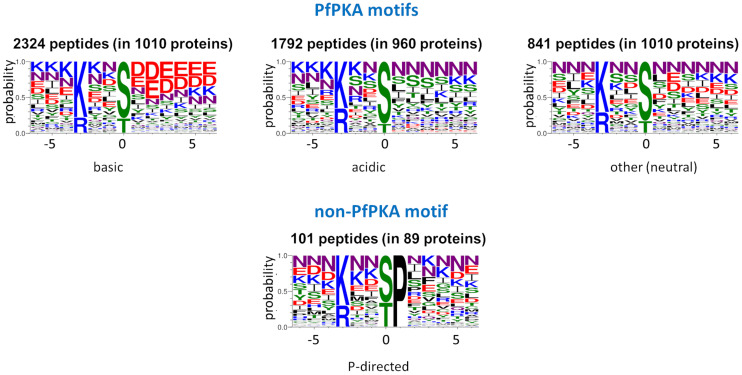
Sequence logos of potential PKA substrate phosphorylation sites. Sequence logos of phosphorylation sites with a basic residue at position –3, extracted from 12 *Plasmodium falciparum* phosphoproteome studies (Treeck et al., 2011; Lasonder et al., 2012, 2015; Pease et al., 2013, 2018; Collins et al., 2014; Alam et al., 2015; Kumar et al., 2017; Flueck et al., 2019; Patel et al., 2019; More et al., 2020), were generated with Web Logo 3 (Crooks et al., 2004) and divided into the acidic, basic and neutral PfPKA motifs and the proline-directed non-PKA motif.

Within the list of 5,735 phosphorylated peptides with the PfPKA core motif, we detected four sequence subclasses (acidic, basic, Pro-directed, and other/neutral) on the basis of the chemical properties of the amino acid side chains (Figure 2). The 101 peptides classified as the Pro-directed subclass were excluded as potential PfPKA substrates because of (1) functional divergence of the proteins from the other subclasses established by a GO enrichment analysis, (2) absence of this motif in known PfPKA substrates, and (3) Pro-directed sequences are classified as cyclin dependent kinase substrates in the Kinexus database comprised of 177,000 human phosphorylation sites^1^. From the remaining 5,634 potential PfPKA phosphorylation sites in 1,675 *P. falciparum* proteins, 5,478 were excluded, because they are in proteins without evidence of a PfPKA interaction in the STRING protein interaction database. This is probably a severe exclusion criterion, because it’s likely that many PfPKA – protein interactions in malaria parasites have yet to be discovered and entered into the STRING database. However, applying this stringent selection resulted in a list of just 156 phosphorylation sites in 30 putative PfPKA substrates (Supplementary Table 3).

In total, we established 109 putative PfPKA – protein substrate relationships from large scale *P. falciparum* protein phosphorylation data sets (Supplementary Table 4). Previously, we have described that protein ubiquitylation dramatically increases as schizonts develop into merozoites (Green et al., 2020) and within the list of 109 putative PKA substrates there are 59 ubiquitylated proteins that we had identified previously in the asexual blood stage ubiquitylome (Green et al., 2020). This is a 5.9-fold enrichment compared to the total *P. falciparum* proteome (*p* = 0.00001). Remarkably, almost all proteins with a PfPKA substrate phosphorylation motif (57) were detected as ubiquitylated in merozoites and heightened phosphorylation of two ubiquitin carboxyl-terminal hydrolases (GeneIDs: PF3D7_0104300, PF3D7_0726500), an E3 ubiquitin-protein ligase (GeneID: PF3D7_1004300) and a ubiquitin-like protein (GeneID: PF3D7_1132000) was observed (Supplementary Table 4).

**FIGURE 3:**
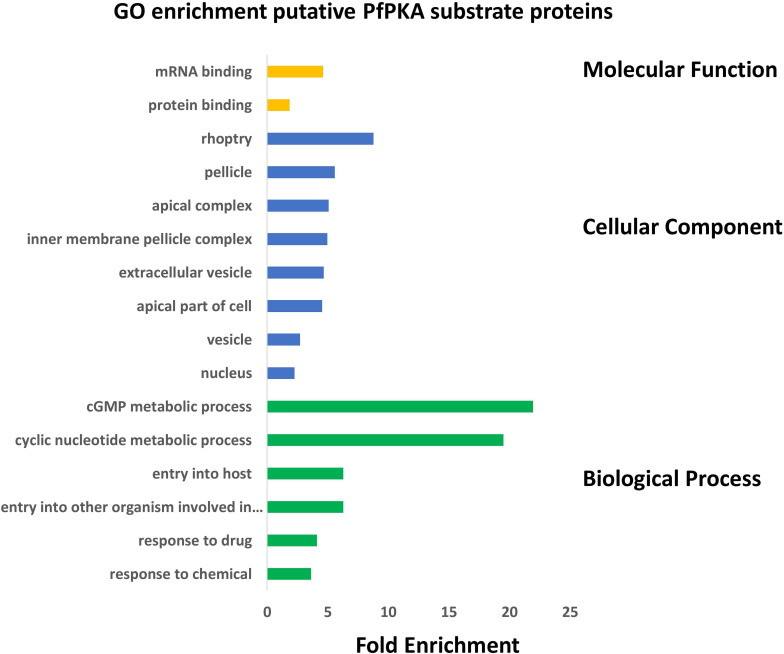
Gene Ontology (GO) enrichment analysis of 109 putative PfPKA substrate proteins. GO enrichment analysis of proposed PfPKA substrates from Supplementary Table 4 was performed with the GO enrichment analysis tool of PlasmoDB (https://plasmodb.org/plasmo/app), and the results are depicted in a histogram for terms with p adjusted (Benjamini-Hochberg) < 0.05. Terms excluded from the histogram are those with less than three PfPKA substrates, as well as very broad terms with more than 2,000 proteins in the *Plasmodium falciparum* proteome.

The biological functions of these putative PfPKA substrates were assessed by a GO enrichment analysis (Figure 3). From this analysis it is clear that PfPKA-mediated phosphorylation is involved in facilitating merozoite invasion of RBCs. Another remarkable feature is the strong influence on cGMP-dependent metabolic processes, as PfPKG has been clearly established as crucial for merozoite egress rather than invasion, where PfPKA plays an essential role (Flueck et al., 2019; Patel et al., 2019). The observation that cGMP phosphodiesterase α (PfPDEα; GeneID: PF3D7_1209500); PfPDEβ and PfPKG are all likely phosphorylated by PfPKA, strongly suggests an interplay between cGMP- and cAMP-signaling processes in intra-erythrocyte parasite development prior to merozoite egress (Flueck et al., 2019), similar to what occurs in mammalian cells (Taylor et al., 2013). However, phosphorylation of these cGMP-signaling enzymes does not increase in extracellular merozoites in EC buffer (Supplementary Table 2). This is consistent with a positive role for PfPKA-mediated regulation of cGMP-PKG-mediated merozoite egress. Moreover, increased phosphorylation of PfACβ and PfPKAc is observed in egressed extracellular merozoites, entirely consistent with the role of cAMP-dependent PfPKA in merozoite invasion of RBCs, established using genetic methodologies (Flueck et al., 2019; Patel et al., 2019).

**FIGURE 4:**
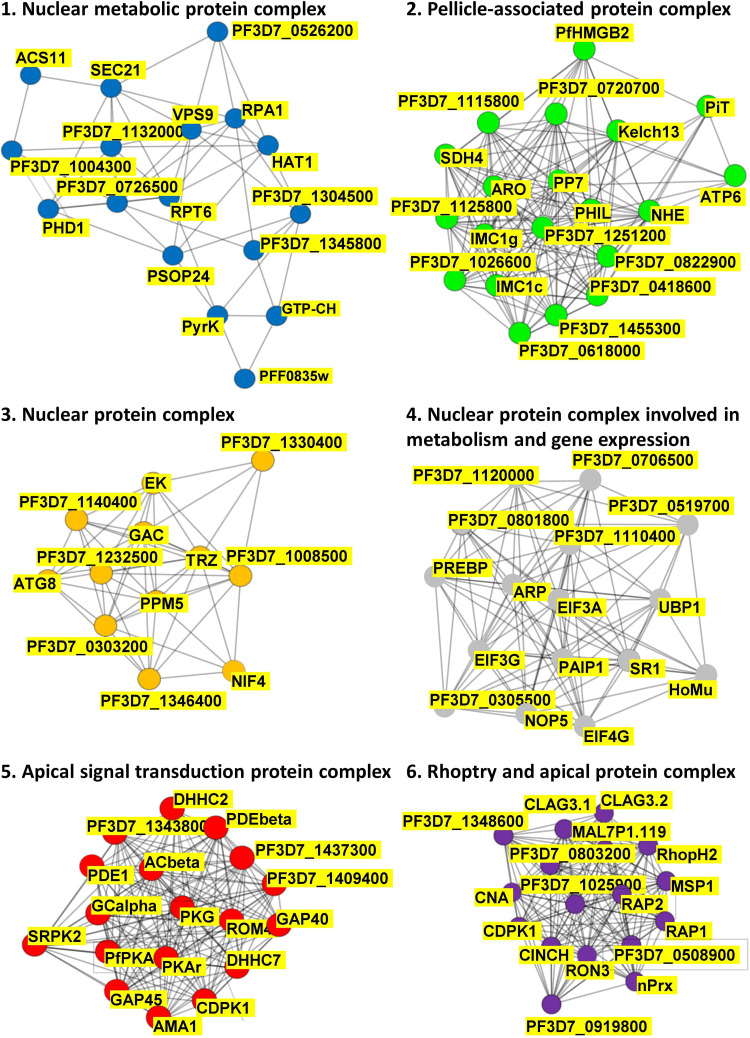
PfPKA-mediated signaling protein interaction networks. Putative protein interaction networks associated with PfPKA signaling were generated by MCL clustering analysis of the PfPKA substrate interactome comprised of 109 proteins (Supplementary Table 4) obtained from the STRING (https://string-db.org/) database of protein–protein interactions. Sub-networks were annotated functionally by Gene Ontology terms on the basis of frequency and enrichment.

The list of 109 putative PfPKA substrate proteins enabled exploration of putative PfPKA-mediated signaling networks by analyzing the protein – protein interactions from the STRING database (Szklarczyk et al., 2019), which resulted in the identification of the six sub-networks depicted in Figure 4 and listed in Supplementary Table 4. Clusters 1, 3, and 4 are comprised of protein complexes localized within the nucleus, with clusters 1 and 4 involved in metabolism and cluster 4 in gene expression. Cluster 2 contains proteins linked to the pellicle, but of largely unknown function. Clusters 5 and 6 are both associated with the apical end of the parasite, and are interconnected via PfCDPK1 that is present in both clusters. Cluster 5 contains PfPKA, PfPDEβ and PfAcβ, the genes that were selected by Patel et al. (2019) for deletion, with analysis of the resultant phosphoproteomes to identify putative PfPKA-targets, as described above. The critical role of cAMP signaling in merozoite invasion is illustrated by eight core proteins from cluster 5 [PfAMA1 (GeneID: PF3D7_1133400), PfCDPK1, PfACβ, PfGCα (GeneID: PF3D7_1138400), PfPDEα, PfPDEβ, PfPKAr, and PfPKAc], and this analysis has been reviewed recently (Perrin et al., 2020). Our data suggest that other proteins from cluster 5 play a role in invasion mediated by PfPKA as well. It is interesting to note that the function of 14 out of the 18 proteins in cluster 5 has been studied by gene disruption and phenotypic analysis in *Plasmodium berghei.* For six proteins, including PfROM4 (GeneID: PF3D7_0506900) and PfDHHC7 (PF3D7_0528400), which are not members of the core of eight proteins in cluster 5, gene disruption resulted in a phenotype in the asexual blood stages. The gene disruption information from the RMgmDB database^2^ for all 109 putative PfPKA substrates is provided in Supplementary Table 4. There are 34 *P. berghei* orthologs (31%) showing a clear phenotype in asexual blood stages following gene disruption.

PfPKA-mediated phosphorylation of Ser610 in the intracellular C-terminal domain of PfAMA1 (Treeck et al., 2009) has been proposed to induce a conformational change that is a necessary late step in successful merozoite invasion (Patel et al., 2019). Substitution of Ser610 abolished the ability of mouse PKA to phosphorylate recombinant PfAMA1, implying that Ser610 is the only PfPKA site in AMA1 (Patel et al., 2019). It was, therefore, surprising that Ser610 was found to be already phosphorylated in merozoites prepared in IC buffer (More et al., 2020). However, increased phosphorylation of Ser590 of PfAMA1 was observed in merozoites in EC buffer compared to IC buffer (Supplementary Table 2). One way to reconcile these contrasting observations is to propose that phosphorylation of S590 occurs in extracellular merozoites only if Ser610 has already been phosphorylated; and, indeed, phosphorylated Ser610 is observed already in non-egressed merozoites in IC buffer (Supplementary Table 2). Perhaps, a conformation change induced by Ser610 phosphorylation (Patel et al., 2019) exposes Ser590 in egressed merozoites, allowing its phosphorylation to lock PfAMA1 in an invasion competent conformation.

We should point out that activating PfPKA may not lead to a systematically higher phosphorylation of PfPKA-target substrate proteins. An interesting example is merozoite surface protein 1 (PfMSP1, GeneID: PF3D7_0930300) with two phosphorylation sites, Ser116 and Ser1582 (Supplementary Table 3), which are potentially significant. Position 116 is in the N-terminal fragment that is present on the merozoite surface, as part of a protein complex. Proteolytic cleavage by PfSUB2 (GeneID: PF3D7_1136900) releases this complex from the merozoite surface during invasion, except for the C-terminal PfMSP1-19 (Blackman et al., 1991). Ser1582 is just upstream of the PfSUB2 cleavage site in a region of 35 amino acid residues that have been proposed to mediate PfMSP1 dimerization on the merozoite surface (Babon et al., 2007). Perhaps when S1582 is phosphorylated PfMSP1 cannot dimerize, but when S1582 is dephosphorylated it can. Phosphorylation of S116 and S1582 was reduced when merozoites were moved from IC to EC buffer, and therefore we looked for protein phosphatases that may be regulated by phosphorylation, for example by upregulation of PfPKA-mediated phosphorylation. We noticed potential PfPKA-mediated phosphorylation of Ser65 in PfPPM5 (GeneID: PF3D7_0810300) and of Ser164 and Ser769 in PfPP7 (GeneID: PF3D7_1423300 – Supplementary Table 3). Interestingly, S769 phosphorylation in PfPP7 increases in extracellular merozoites (More et al., 2020) and it is possible that phosphorylation increases the phosphatase activity of PfPP7, leading to dephosphorylation of Ser116 and Ser1582 of MSP1, and this promotes merozoite invasion. Another potential PfPKA-target residue is Ser106 in rhoptry associated protein 1 (PfRAP1, GeneID: PF3D7_1410400); phosphorylation of this residue also decreased in EC buffer, suggesting that dephosphorylation of PfRAP1 Ser106 is also mediated by either PfPPM5 or PfPP7. In contrast, while phosphorylation of S106 decreased, the level of PfRAP1 phosphoSer183 increased, clearly indicating that in extracellular merozoites the putative PfPKA-mediated phosphorylation of a given protein (in this case PfRAP1) can both increase and decrease, depending on the particular target phosphosite. As described above, another example of the complexity of phospho-regulation is PfAMA1, where phosphorylation of S590 increases, while the phosphorylation status of S610 remains unchanged, in extracellular merozoites.

There are 16 putative PfPKA-target substrate proteins that display increased phosphorylation at one or more sites, and two substrate proteins whose phosphorylation decreases, when merozoites are placed in EC extracellular buffer (listed in Supplementary Table 2). For PfPKA, PfPPM5 and PfPP7 to be responsible for altered phosphorylation of putative PfPKA-target proteins they have to be in (transient) physical contact with their substrates, and therefore caution is necessary, as we do not know the subcellular compartments of PfPPM5 and PfPPM7. The potential of all 18 proteins as therapeutic targets cannot be discussed here, but clearly their possible role in merozoite invasion, modulated by altered phosphorylation, is worthy of detailed studies, as is the link between heightened PfPKA-mediated phosphorylation and ubiquitylation in extracellular merozoites. These substrates can now be investigated, for example by genetic approaches, including identifying the importance of individual phosphorylation sites, using CRISPR-Cas9 technology.

## Targeting Host PKA to Inhibit Different Stages of the *Plasmodium* Life Cycle and Inhibit Other Apicomplexan Infections

The role of cAMP/PKA signaling in invasion of erythrocytes by *Plasmodium* merozoites is well known, as described above. Pharmacological inhibitors of mammalian cAMP regulatory and responsive proteins can interfere with invasion (Syin et al., 2001; Salazar et al., 2012). Furthermore, disruption of PfACβ (Patel et al., 2019), PfPKAc (Patel et al., 2019; Wilde et al., 2019) and PfPDEβ (Flueck et al., 2019) renders merozoites unable to invade RBCs (Patel et al., 2019; Wilde et al., 2019). Infection of RBC by *P. falciparum* leads to a rise in intra-erythrocyte cAMP levels (Syin et al., 2001; Merckx et al., 2008; Dawn et al., 2014; Ramdani et al., 2015), activating both host and parasite PKA and triggering a phosphorylation cascade of both host (Bouyer et al., 2016) and parasite (Lasonder et al., 2012, 2015) proteins, and contributing to malaria pathogenesis.

However, cAMP signaling also plays an important role at other stages in the parasite’s life cycle, for example in hepatocyte invasion by *Plasmodium* sporozoites and gametocyte transmission. cAMP is involved in sporozoite exocytosis through the regulation of intracellular Ca^2+^ levels. *P. berghei* parasites deficient in adenylyl cyclase α (PbACα, GeneID: PBANKA_1037500) no longer exocytose during migration through host cells, and lose hepatocyte infectivity *in vivo* (Ono et al., 2008). cAMP-signaling is also involved in gametocyte-infected erythrocyte (GIE) deformability. Reduced levels of cAMP render stage V gametocytes less rigid and hence less likely to be trapped and cleared by the reticuloendothelial system in the spleen. Raising cAMP levels via pharmacological inhibition of phosphodiesterases renders stage V gametocytes more rigid, and therefore use of phosphodiesterase inhibitors has been proposed as a strategy to block transmission of malaria parasites (Ramdani et al., 2015).

Use of reverse phase protein microarray technology demonstrated that *Plasmodium yoelii* infection impacts hepatocyte regulatory pathways involved in cell survival, proliferation, and autophagy. Decreased pro-apoptotic phosphorylation of BAD (B-cell lymphoma [Bcl]-2 associated death promotor) and increased levels of the apoptosis inhibitor Bcl2 likewise contribute to an anti-apoptotic environment promoting survival of the parasite in its host (Kaushansky et al., 2013). It is known that Ser155 in BAD is preferentially phosphorylated by PKA (Lizcano et al., 2000) and *P. falciparum* infection leads to phosphorylation of a variety of cytosolic host erythrocyte proteins, including BAD (Carvalho et al., 2016). This suggests that cAMP-PKA signaling may also interfere with an erythrocyte Bcl-dependent apoptosis mechanism to protect against infected red blood cell death.

There is evidence that a prostaglandin-cAMP-PKA signaling pathway may contribute to the pathogenesis of *P. falciparum* asexual blood stages. Prostaglandins are known to modulate cAMP concentrations and *P. falciparum*-infected RBCs produce prostaglandins that may contribute to malaria-associated pathology (Kilunga Kubata et al., 1998). For instance, induction of heme oxygenase-1 expression by prostaglandin D(2) may be involved in the pathogenesis of cerebral malaria (Kuesap and Na-Bangchang, 2010). In addition, decreased cyclooxygenase-2 (COX2)-derived prostaglandin E2 (PGE2) levels enhanced clinical severity in cerebral malaria (Perkins et al., 2001). In addition, suppression of COX-2-derived PGE2 is associated with reduced erythropoiesis and worsening anemia in children with falciparum malaria (Anyona et al., 2012).

As in *Plasmodium* infection, cAMP-signaling contributes to the pathogenesis of the related apicomplexan parasites *Theileria* and *Toxoplasma*. In *Theileria*-infected and transformed leukocytes, surges in cAMP concentration trigger a myriad of cellular responses via activation of both PKA and EPAC (exchange protein activated by cAMP) and their downstream effectors. EPAC is a mediator of cAMP signaling distinct from PKA and has two isoforms, EPAC1 and EPAC2, which are also known as RAP (Ras-related protein) guanine nucleotide exchange factors 3 and 4 (de Rooij et al., 1998; Kawasaki et al., 1998). Higher levels of TGF-β2 stimulate *COX2* and Prostaglandin E Receptor 4 (EP4) expression in virulent infected macrophages leading to a PGE2 auto-stimulatory loop that modulates cAMP levels. Blocking PGE2-signaling decreased cAMP levels, while activation of PGE2/EP4 signaling increased production of cAMP and stimulated adhesion and Matrigel traversal (Haidar et al., 2015). A similar role for PGE2 signaling in modulating cAMP levels has been reported for HIV-infected macrophages (Hayes et al., 2002). Also, cAMP regulates cytosolic Ca^2+^ levels via induction of an EPAC/CaMKII/CREB pathway leading to an increase in dissemination of *Theileria*-infected macrophages (Haidar et al., 2015, 2017). Hexokinase-2 (HK2) binds to BAD only when serine 155 in BAD is phosphorylated by PKA. Dissociation of HK2/BAD complex by a penetrating peptide that acts as a non-phosphorylatable BAD substrate provokes ubiquitination and degradation of HK2 by the proteasome. Loss of HK forces *Theileria-*transformed macrophages to switch from HK2-dependent Warburg glycolysis to HK1-dependent oxidative glycolysis, which dampens macrophage proliferation (Haidar et al., 2017). Finally, cAMP fluxing impacts on the virulence of *Theileria*-transformed leukocytes through modulation of H_2_O_2_ output (Haidar et al., 2019). cAMP induces CREB transactivation to stimulate *catalase* transcription leading to increased catalase activity and reduced H_2_O_2_ output (Haidar et al., 2019).

As in *Theileria* and *Plasmodium* infection, cAMP-signaling plays a critical role in the pathogenesis of *Toxoplasma.* For example, cAMP can have both stimulatory and antagonistic effects on bradyzoite differentiation. A transient raise in cAMP level promotes bradyzoite differentiation, whereas its prolonged elevation inhibits this process (Kirkman et al., 2001). Moreover, cAMP contributes to the neuropathology of *Toxoplasma* infection by inducing CREB transactivation to drive miR-132 expression that downregulates D1-like dopamine receptors and hence dopamine signaling (Xiao et al., 2014). Additionally, pharmacological inhibition of PDE activity could be a promising strategy for treatment of toxoplasmosis. Rolipram, a PDE4 inhibitor interferes with the transition of *Toxoplasma* to a chronic phase of infection by increasing levels of intracellular cAMP, thus, preventing biochemical and histological signs of *Toxoplasma*-induced hepatitis in mice, as well as brain pathology of latent toxoplasmosis (Afifi and Al-Rabia, 2015). Also, PDE4 inhibitors can modulate the inflammatory response affecting conversion to bradyzoites and pathological patterns of *Toxoplasma* infection (Lyons et al., 2002; Sullivan and Jeffers, 2012).

TgPKA is involved in calcium-signal transduction in *Toxoplasma* (Lourido et al., 2010). cAMP fluxes likely activate TgPKAc1 (GeneID: TGGT1_242070) that stimulates microneme secretion and tachyzoite egress from host cells (Lourido et al., 2010). Furthermore, TgPKA is involved in stage interconversion of tachyzoite to bradyzoite asexual stages of *T. gondii* mainly via the catalytic subunit TgPKAc3 that is responsible for maintenance of the tachyzoite stage during infection (Sugi et al., 2016). Additionally, overexpression of TgPKAc1, or conditional depletion of TgPKAr, leads to a defect in intracellular growth (Jia et al., 2017).

**FIGURE 5:**
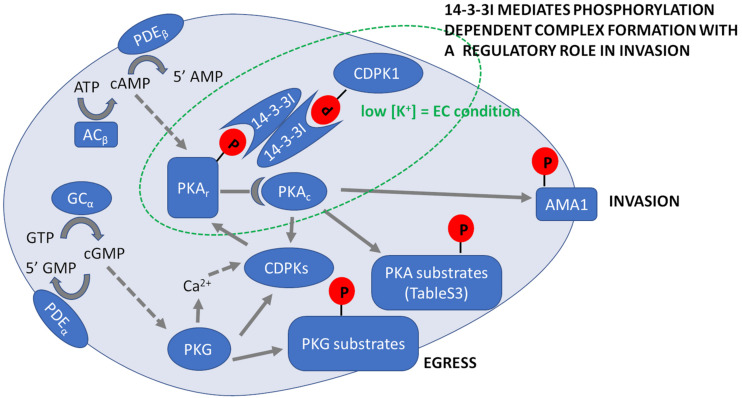
Summary of *Plasmodium falciparum* cyclic nucleotide signaling during erythrocyte invasion. Prior to merozoite egress PKG is activated by binding cGMP, which is produced by the conversion of GTP to cGMP by GC. Activation of PKG induces an increase in intracellular Ca^2+^ and activation of calcium-dependent kinases (CDPKs), which leads to secretion of apical proteins leading to egress of merozoites from mature schizonts. Egressed merozoites in extracellular ionic conditions (EC) with low K^+^ levels activate ACβ that converts ATP to cAMP. PKAr, the regulatory subunit of PKA binds cAMP releasing the catalytic subunit PKAc to phosphorylate specific target substrates that are listed in Supplementary Table 3. In extracellular merozoites PKA phosphorylates S590 in the cytoplasmic tail of AMA1 and we hypothesize it locks AMA1 in an invasion competent conformation provoked by PKA-mediated S610 phosphorylation of AMA1 prior to merozoite release from red blood cells. Following merozoite egress, phosphorylation of both PKAr and CDPK1 increases to promote their binding to 14-3-3I, so forming a multi-protein complex mediating secretion of microneme proteins such as AMA1 required for invasion. Phosphorylation of targets by various kinases is indicated by solid arrows. Activation of kinases by second messengers is indicated with dotted arrows. Cyclic nucleotides are hydrolyzed by PDEα and PDEβ.

Overall, there are multiple examples of how cAMP signaling contributes to the pathogenesis of different apicomplexan parasites. This argues that pharmacological manipulation of cAMP levels and blockade of PKA-mediated signaling could be an effective therapeutic strategy in the fight against disease caused by apicomplexan parasites. In Figure 5 we present a scheme for malaria parasites that highlights the different enzymes involved, all of which are potential therapeutic targets.

## Author Contributions

All authors listed have made a substantial, direct and intellectual contribution to the work, and approved it for publication.

## Conflict of Interest

The authors declare that the research was conducted in the absence of any commercial or financial relationships that could be construed as a potential conflict of interest.

## Footnotes

1 http://www.kinexus.ca/

2 https://www.pberghei.eu/

## Supplementary Material

Supplementary Table 1 High confidence PfPKA substrate phosphorylation sites from Patel et al. (2019) and presence of PKA phosphorylation motif.

Supplementary Table 2 Putative PfPKA substrate phosphorylation sites from More et al. (2020) and presence of PKA phosphorylation motif.

Supplementary Table 3 Potential PfPKA substrate phosphorylation sites from 12 *P. falciparum* phosphoproteome studies with presence of core PKA phosphorylation motif and interaction with PKA.

Supplementary Table 4 Putative PfPKA substate proteins from Supplementary Tables 1–3 with protein interaction networks from STRING and gene disruption phenotypes from RMgmDB.
